# Tracheal, bronchus, and lung cancer among older adults: thirty-year global burden trends, precision medicine breakthroughs, and lingering barriers

**DOI:** 10.1186/s12885-025-14363-x

**Published:** 2025-05-28

**Authors:** Hongquan Xing, Cong Wu, Weichang Yang, Shanshan Cai, Xinyi Zhang, Xiaoqun Ye

**Affiliations:** 1 Department of Respiratory and Critical Care Medicine, The Second Affiliated Hospital, Jiangxi Medical College, Nanchang University, Nanchang, 330006 China; 2 Jiangxi Key Laboratory of Molecular Medicine, The Second Affiliated Hospital of Nanchang University, Nanchang, 330006 China; 3 Department of Pathology, The Second Affiliated Hospital, Jiangxi Medical College, Nanchang University, Nanchang, 330006 China

**Keywords:** Tracheal bronchus and lung cancer, Aging population, Environmental particulate pollution, Precision therapy, Disability-adjusted life years

## Abstract

**Background:**

Tracheal, bronchial, and lung (TBL) cancer presents significant health challenges for individuals aged 70 and older. However, comprehensive insights into the epidemiological patterns of and risk factors for TBL cancer in this population remain limited. This study aimed to analyze the global, regional, and national burdens and trends of TBL cancer patients aged ≥ 70 years from 1990–2021.

**Methods:**

The incidence, mortality, and disability-adjusted life years (DALYs) for TBL cancer patients aged ≥ 70 years from 1990–2021 were obtained from the 2021 Global Burden of Disease study. Global trends were stratified age, sex, and sociodemographic index (SDI). Decomposition analysis identified the primary drivers of burden changes, and a global risk attribution analysis was conducted. The Bayesian Age‒Period‒Cohort (BAPC) model forecasted trends over the next 14 years. The analyses were performed with Joinpoint software and the R software.

**Results:**

From 1990–2021, the ASIRs, ASMRs, and ASDRs of TBL cancer among patients ≥ 70 years increase significantly, mainly due to aging and population growth. In the precision medicine era (2015–2021), these indicators for both sexes and males have declined, but the burden among females has increased. The burden varies across regions, with the incidence of TBL cancer increasing more severely in middle-SDI regions, East Asia, and western sub-Saharan Africa, whereas high-SDI regions have shown a decline after peaking. Although the DALY proportion of smoking decreased, it was still the main cause of TBL cancer. However, the burden of environmental particulate pollution has increased. The BAPC model predicted that in the future, the ASIR, ASMR, and ASDR for males and both sexes would decrease, whereas these indicators would either remain stable or increase among females.

**Conclusions:**

The burden of TBL cancer is increasing significantly among patients aged ≥ 70 years. Despite new hopes and approaches from precision medicine, environmental and behavioral factors still critically influence the TBL cancer burden. Future strategies could enhance subgroup-specific management and promote effective control of known risk factors.

**Supplementary Information:**

The online version contains supplementary material available at 10.1186/s12885-025-14363-x.

## Introduction

Tracheal, bronchial, and lung (TBL) cancer is predominantly an age-related disease that is influenced by factors such as environmental exposure (e.g., tobacco, air pollutants); more than 40% of cases occur in patients aged 70 years and older, thus posing significant societal, public health, and economic challenges [1, 2]. Although international studies typically use 65 years as the cutoff age for geriatric assessment, the European Organization for Research and Treatment of Cancer (EORTC) and the International Society of Geriatric Oncology (SIOG) have selected 70 years as the threshold age, and this standard is widely used in clinical trials [3, 4]. As the global population ages at an increasingly rapid rate, the proportion of TBL cancer cases among this age group is expected to increase further [5]. Currently, treating elderly patients with TBL cancer poses a significant clinical challenge. With advancing age, patients frequently develop multiple comorbidities and organ dysfunction, which can directly impact the absorption, distribution, metabolism, and excretion of drugs, ultimately diminishing their treatment tolerance [6]. Previous studies have indicated that patients aged over 70 years have a substantially shorter life expectancy, with a 5-year survival rate ranging from only 9% to 15%, compared with rates ranging from 18%—28% among those under 70 years [7, 8].

Precision medicine can provide targeted and personalized treatments on the basis of patients’ unique genetic, molecular, and clinical characteristics. In 2003, the Food and Drug Administration (FDA) approved gefitinib as the first targeted drug for non-small cell lung cancer (NSCLC). Since 2004, its extensive application in clinical practice has marked the beginning of the precision medicine era in lung cancer treatment [9]. The development of new targeted drugs and immunotherapies, along with advancements in surgical procedures, has subsequently offered more effective treatment options for elderly patients with TBL cancer [10–12]. Although these treatment methods have brought new treatment opportunities to older patients with TBL cancer, some problems remain in terms of practical application. The proportion of elderly patients receiving treatment is decreasing [13, 14]. Moreover, owing to the combined influence of various social, economic, and family factors, there are also significant differences among older patients with lung cancer in different countries and regions in terms of their participation in molecular testing and treatment [15]. Previous studies among elderly lung cancer patients have focused mainly on precision medicine-based clinical treatment and individualized management (e.g., clinical trials and case studies [16, 17]). There might be insufficient representation of elderly individuals in clinical trials [18], and little attention has been devoted to their global epidemiological features and disease burden in the precision medicine era.

Consequently, this study analyzed the burden of TBL cancer among elderly patients in 204 countries and regions during the development process of precision medicine from 1990 to 2021 by analyzing data from the 2021 Global Burden of Disease (GBD). These data offer valuable epidemiological insights by detailing global risk factor exposure and associated disease burdens by age, sex, cause, and location [19]. Additionally, we examined the modifiable risk factors for TBL cancer in the older population across varying sociodemographic index (SDI) levels and predicted the future trends of TBL cancer among older adults until 2035. Our goal is to offer valuable guidance for optimizing global health resource allocation, enhancing awareness, and improving responses to older TBL cancer patients, thus addressing the challenges faced by the aging global population.

## Materials and methods

### Data sources

We extracted data on the incidence rate, mortality rate, disability-adjusted life years (DALYs), and risk factors for TBL cancer in individuals aged ≥ 70 years from the GBD 2021 database (https://vizhub.healthdata.org/gbd-results/) for the period 1990–2021. Based on revolutionary breakthroughs in treatment modalities, the modern treatment history of TBL cancer can be classified into three stages. The chemotherapy-dominated era (1990–2003) focused on platinum-based drugs and lacked personalized strategies. The targeted-therapy breakthrough era (2004–2014) started with the widespread use of gefitinib in 2004, which promoted driver gene-based personalized treatment [9]. The precision immunotherapy era (2015–2021) was marked by the FDA’s approval of a PD-1 inhibitor (nivolumab) in 2015. Subsequently, clinical research on immunotherapy combinations has flourished, thereby expanding treatment options, improving the disease prognosis, and establishing a biomarker-guided multidimensional treatment system [20].

The dataset included mean values and 95% uncertainty intervals (UIs) stratified by age groups (70–74, 75–79, 80–84, 85–89, 90–94, and 95 + years), SDI regions, 21 GBD global regions, and 204 countries. Grouping age into intervals allows studies to calculate age-adjusted disease risks and mortality rates, offering a more accurate picture of disease prevalence trends and burdens. This approach aligns with public health and clinical research practices, thereby ensuring comparability with other studies and databases [21]. As indicated in Table S1, the TBL cancer coding followed the ICD-10 classification. Variables were selected to comprehensively assess disease burden and socioeconomic inequalities, with the SDI serving as a proxy for socioeconomic and public health conditions. To evaluate the attributable burden of risk factors associated with TBL cancer, we extracted data on 16 detailed risk factors that were automatically matched to TBL cancer from the GBD 2021 study. Detailed definitions and classifications of these risk factors are available on the website https://www.healthdata.org/research-analysis/health-risks-issues [22].

### Statistical analysis

We calculated age-standardized incidence rates (ASIRs), mortality rates (ASMRs) and DALYs (ASDRs) for TBL cancers on the basis of a global standard population (GBD Standard Population Age Distribution) using direct standardization methods. Detailed descriptions can be found in other studies [19, 23]. Temporal trends from 1990–2021 were evaluated using the average annual percentage change (AAPC), which was calculated with Joinpoint software (version 5.2.0). The AAPC provides a summary of these trends over specific periods as a weighted average of annual percentage changes (APCs). The specific calculation methods and descriptions can be found in a previous study [24]. Future projections (2022–2035) were made using a Bayesian Age-Period-Cohort (BAPC) model. With respect to the projections, previous studies have shown that the predictive performance of this model is excellent, and the specific methods have been described in previous studies [23, 25]. The results are expressed per 100,000 people with 95% UIs. All the statistical analyses and data visualization were performed via the R software package (version 4.2.3). For trend analyses, *p* values < 0.05 were considered statistically significant.

## Results

### Global trends in the burden of tracheal, bronchial, and lung cancer among older people

**Fig. 1:**
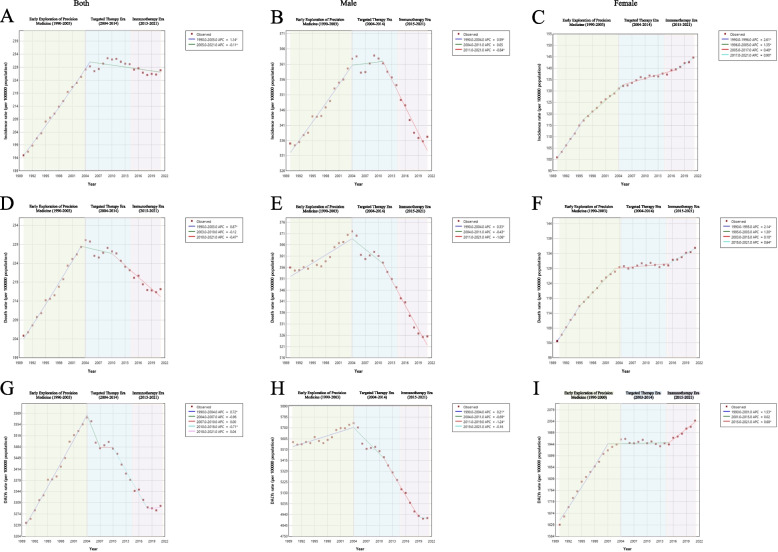
Joinpoint regression analysis of the ASIR, ASMR and ASDR of global TBL cancer in older patients (aged 70 years and older) from 1990 to 2021. ASIRs: **A** both sexes, **B** males, **C** females; ASMRs: **D** both sexes, **E** males, **F** females; ASDRs: **G** both sexes, **H** males, **I** females. Abbreviations: ASIRs = age-standardized incidence rates, APC = annual percentage change, ASMRs = age-standardized mortality rates, DALYs = disability-adjusted life years, ASDRs = age-standardized rate of DALYs, AAPC = average annual percent change, TBL = tracheal, bronchial, and lung

From 1990–2021, the AAPCs in the ASIR, ASMR, and ASDR of TBL cancer among the global elderly population were 0.49 (95% CI: 0.44–0.54), 0.17 (95% CI: 0.10–0.24), and 0.05 (95% CI: 0.02–0.07), respectively (Tables S2, S3 and S4). Joinpoint regression analysis indicated that the ASIR of elderly patients with TBL-related cancer demonstrated a statistically significant upward trajectory from 1990 to 2005. However, from 2005 onward, the ASIR exhibited a sustained decline (Fig. 1A). For the ASMR, a significant increase was observed between 1990 and 2003, followed by stabilization from 2003 to 2010 and a progressive decline after 2010 (Fig. 1D). The ASDR changes paralleled those of the ASMR, as shown in Fig. 1G.

From 1990–2021, the ASIR, ASMR, and ASDR of TBL cancer in male patients remained stable or declined, whereas the rates in female patients exhibited significant increasing trends (Fig. 1). The AAPCs for females were 1.17 (95% CI: 1.09–1.24) for ASIR, 0.84 (95% CI: 0.78–0.91) for ASMR, and 0.68 (95% CI: 0.60–0.75) for ASDR. From 1990 to 2004, males and females presented increasing trends in all three indicators. However, from 2004–2010, male patients exhibit stable or declining trends (ASIR: APC = 0.05, *P* > 0.05; ASMR: APC = −0.43, *P* < 0.05; ASDR: APC =  − 0.69, *P* < 0.05), whereas rates continued to rise among females. After 2015, the differences between male and female patients further widened. Male patients demonstrated accelerated declines across all the metrics (ASIR: APC =  − 0.84; ASMR: APC =  − 1.06; ASDR: APC =  − 1.24; all *P* < 0.05). Conversely, female patients maintained upward trajectories (ASIR: APC = 0.90; ASMR: APC = 0.64; ASDR: APC = 0.68; all *P* < 0.05), highlighting persistent gender disparities in TBL cancer epidemiology.

Global trend analysis between 1990 and 2021 revealed a significant increase in the TBL cancer ASIR among those aged 70 years and above, with the largest increase observed in people aged 95 + years (Table S5). Moreover, the ASMR and ASDR of TBL lung cancer in the population aged 80 years and above also increased, with the most notable increase in those aged 95 years and above, where the AAPC values were 1.70 (95% CI: 1.65–1.74) and 1.64 (95% CI: 1.60–1.68), respectively. In contrast, these two indicators tended to decrease in the 70–74-year-old and 75–80-year-old cohorts.

### Global trends by the SDI

**Fig. 2:**
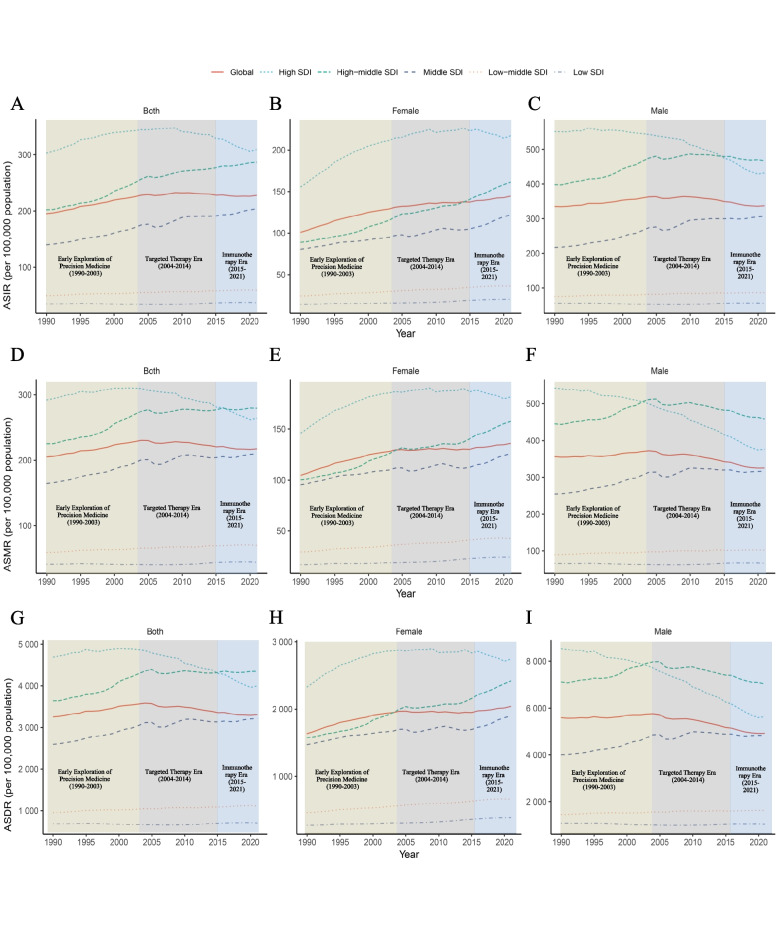
Temporal trends in the ASIR, ASMR and ASDR of global TBL cancer in older patients (aged 70 years and older) from 1990 to 2021 in different SDI regions. ASIRs for **A** both sexes, **B** males, **C** females; ASMRs for **D** both sexes, **E** males, **F** females; and ASDRs for **G** both sexes, **H** males, **I** females. Abbreviations: ASIRs = age-standardized incidence rates, ASMR = age-standardized mortality rates, DALYs = disability-adjusted life years, ASDRs = age-standardized rate of DALYs, SDI = sociodemographic index, TBL = tracheal, bronchial, and lung

Figure 2 illustrates the 1990–2021 temporal trends of the ASIR, ASMR, and ASDR for TBL cancer among elderly patients across SDI regions. High-SDI regions maintained a globally dominant ASIR throughout the study period (peak: 308.91/100,000 in 2021) but demonstrated a mortality paradox. During the targeted therapy era (2004–2014), the ASMR trajectory reversed dramatically from a pre-2004 slow increase (AAPC = 1.49, 95% CI: 1.33–1.65) to a steep decline (AAPC =  − 2.25, 95% CI: − 2.54 to − 1.96). This downward momentum intensified in the precision immunotherapy era (2015–2021), while high-middle-SDI regions emerged as the 2021 ASMR epicenter (279.45/100,000). Conversely, middle-SDI regions exhibited the most rapid increase across all metrics, with the highest AAPCs for ASIR (1.26, 95% CI: 1.18–1.34), ASMR (0.79, 95% CI: 0.68–0.90), and ASDR (0.70, 95% CI: 0.59–0.80).

Among male patients, the ASIR, ASMR, and ASDR across different SDI regions exhibited significant temporal and regional variations. During the chemotherapy-dominated era (1990–2003), high-SDI regions demonstrated the highest ASIRs. With the transition to the targeted therapy era (2004–2014), the ASIR declined markedly. The emergence of precision immunotherapy (2015–2021) further accelerated this downward trend in ASIR. Notably, during the immunotherapy era, high-middle-SDI regions surpassed high-SDI regions in terms of the ASIR (Fig. 2C). A parallel pattern was observed for mortality and disability rates: in the targeted therapy era, high-middle-SDI regions replaced high-SDI regions with the highest ASMRs and ASDRs (Fig. 2F and I).

Among female patients in the high-SDI region, the ASIR, ASMR, and ASDR remained at peak levels across all three therapeutic phases (Fig. 2B, E and H). Upon entering the precision immunotherapy era (2015–2021), this region exhibited significant declines in the ASIR, ASMR, and ASDR, with AAPCs of −0.71 (95% CI: −1.24 to −0.15; *p* = 0.012), −0.67 (95% CI: −1.29 to −0.06; *p* = 0.032), and −0.80 (95% CI: −1.39 to −0.17; *p* = 0.018), respectively. In contrast, all other SDI regions demonstrated increasing trends in these epidemiological metrics during the same period. Notably, the middle-SDI region experienced the steepest increases in the ASIR, ASMR, and ASDR, particularly in the precision immunotherapy era (2015–2021), with AAPCs of 2.94 (95% CI: 2.68–3.19; *p* < 0.001), 2.26 (95% CI: 2.00–2.52; *p* < 0.001), and 1.95 (95% CI: 1.78–2.13; *p* < 0.001), respectively.

**Fig. 3:**
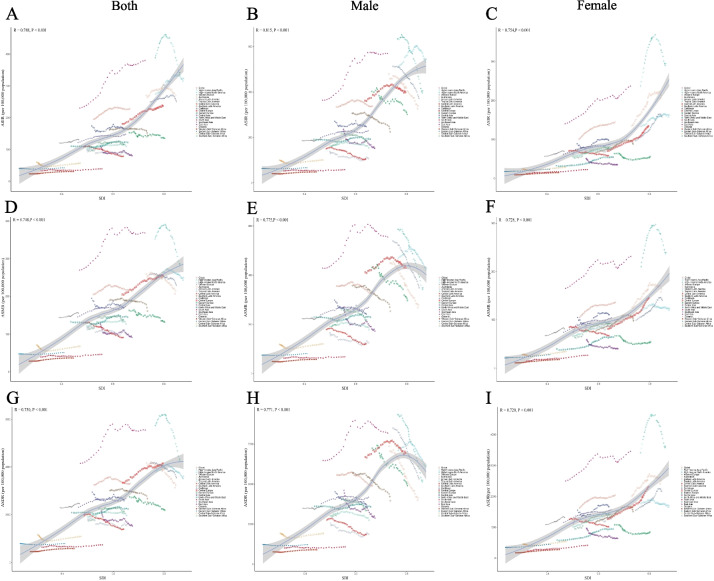
Association between older TBL cancer patients (aged 70 years and older) and the ASIR, ASDR, and ASMR with SDI at the regional level from 1990 to 2021. The associations between regional—level SDI and ASIRs (**A**: both sexes, **B**: males, **C**: females), ASMRs (**D**: both sexes, **E**: males, **F**: females), and ASDRs (G: both sexes, H: males, I: females) of older TBL patients. Abbreviations: ASIRs = age-standardized incidence rates, ASMRs = age-standardized mortality rates, DALYs = disability-adjusted life years, ASDRs = age-standardized rate of DALYs, SDI = sociodemographic index, TBL = tracheal, bronchial, and lung

As shown in Fig. 3, the ASIR, ASDR, and ASMR progressively increased as SDI values increased among both sexes and among females. In contrast, males demonstrated an initial upward trend followed by a significant decline in the ASIR, ASDR, and ASMR at higher SDI values (Fig. S1). At the national level, the SDI was strongly and positively correlated with the ASIR, ASMR, and ASDR across all groups (R = 0.79 for both sexes, 0.75 for females, and 0.75 for males; Fig. S1). Nevertheless, the AAPC displayed an inverse relationship, generally decreasing as the SDI increased (Fig. S2).

**Fig. 4:**
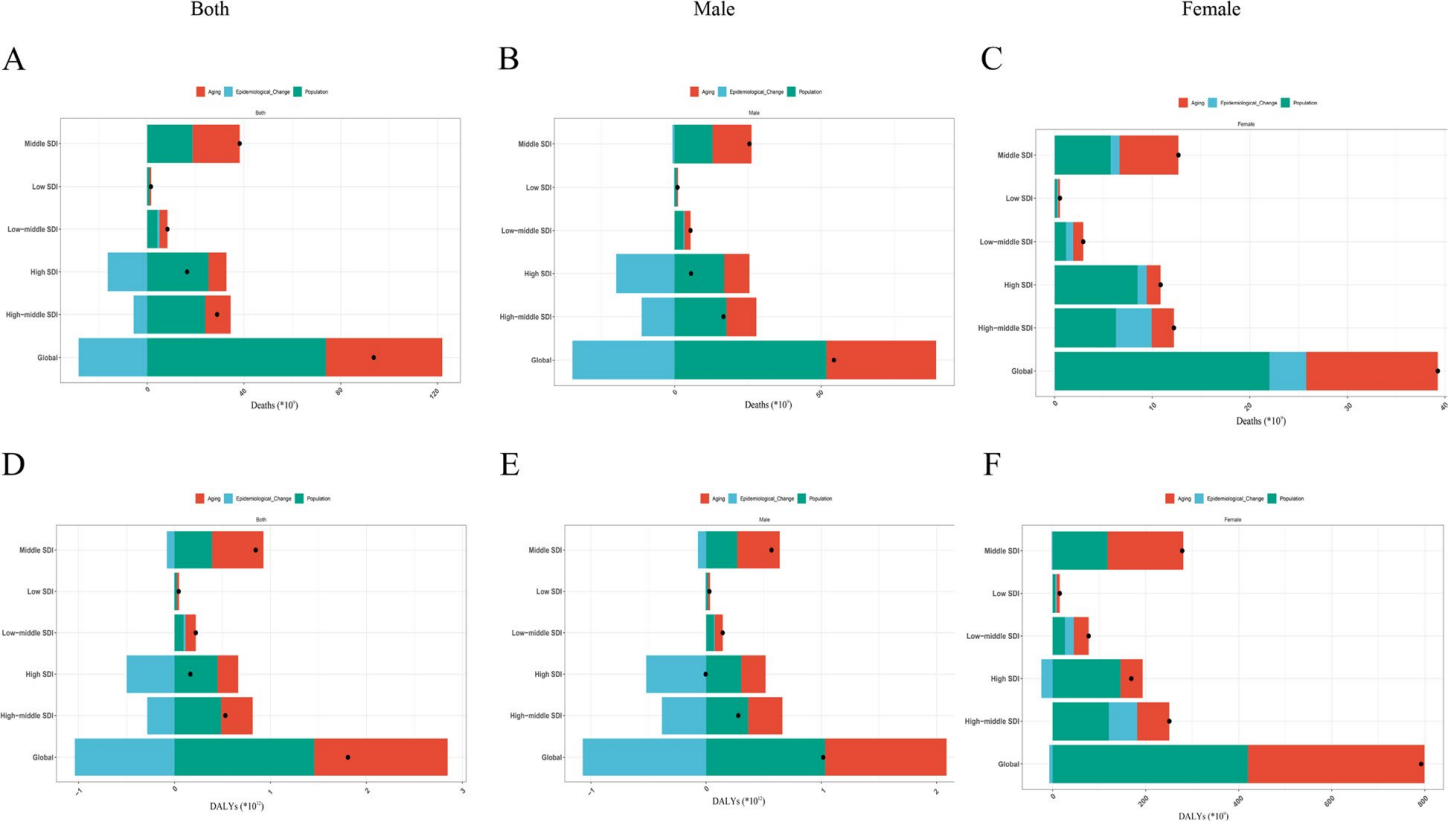
Decomposition analysis by global population-level determinants of changes in the deaths and DALYs of TBL cancer patients stratified by SDI levels and sex from 1990 to 2021. Changes in deaths stratified by SDI in **A** both sexes, **B** males, and **C** females; and changes in DALYs in **D** both sexes, **E** males, and **F** females. The global population-level determinants include three components, aging (in red), epidemiological change (in blue), and population (in green), for which the magnitude of a positive value indicates a corresponding increase in deaths or DALYs of TBL cancer patients attributed to the component, whereas the magnitude of a negative value indicates a corresponding decrease in deaths or DALYs of TBL cancer patients attributed to the related component. The black dots represent the overall values of the changes contributed by all three components. Abbreviations: DALYs = disability-adjusted life years, SDI = sociodemographic index, TBL = tracheal, bronchial, and lung

On the basis of the decomposition analysis, from 1990–2021, the number of deaths and DALYs from older TBL cancer in global SDI regions showed an increasing trend. Particularly in regions with middle SDI values, the increase in disease burden was most significant, with aging being the primary driving factor, followed by population growth. Globally, the negative impact of the epidemiological transition was most pronounced in the regions with the highest quintile of SDI (Fig. 4A and D). Gender difference analysis revealed that this transition exhibited significant gender disparities worldwide: male contributions to deaths and DALYs significantly declined, whereas female contributions increased. DALYs decreased only in high-SDI areas, which was driven primarily by the negative impact of the male epidemiological transition (Fig. 4B, C, E, and F).

### Regional and national trends

**Fig. 5:**
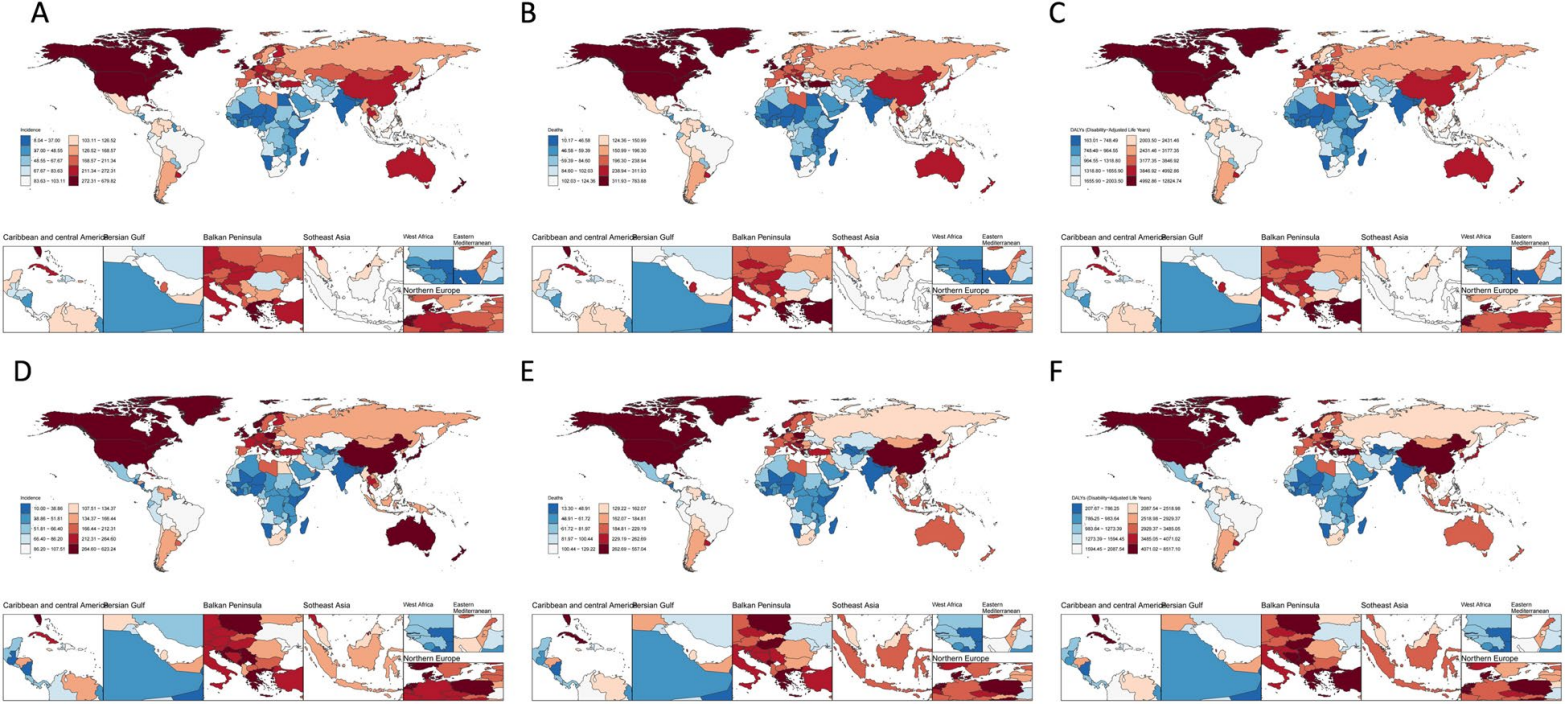
The global distribution of the ASIR, ASMR, and ASDR of TBL cancer in older patients (aged 70 years and older) in 1990 and 2021. Global maps of ASIR (**A**), ASMR (**B**), and ASDR (**C**) in 1990; and global maps of ASIR (**D**), ASMR (**E**), and ASDR (**F**) in 2021 for both sexes. Abbreviations: ASIR = age-standardized incidence rate, ASMR = age-standardized mortality rate, ASDR = age-standardized rate of DALYs, DALYs = disability-adjusted life years, TBL = tracheal, bronchial, and lung

Among the 21 regions covered by the GBD study, the ASIR, ASMR, and ASDR increased most rapidly in East Asia and western sub-Saharan Africa from 1990 to 2021, whereas the most significant declines occurred in high-income North America and Central Latin America (Fig. S3). Gender disparities were evident: males consistently presented higher ASIRs, ASMRs, and ASDRs than females did. However, males also experienced steeper declines in these metrics. In high-income North America, the AAPCs for males (1990–2021) reached −1.59 (95% CI: −1.84, −1.33) for ASIR, −1.82 (95% CI: −2.03, −1.61) for ASMR, and −2.01 (95% CI: −2.23, −1.80) for ASDR. Conversely, women in North Africa and the Middle East experienced substantial increases, with AAPCs of 2.20 (95% CI: 2.02 to 2.38) for ASIR, 2.12 (95% CI: 1.93 to 2.30) for ASMR, and 1.99 (95% CI: 1.84 to 2.14) for ASDR (Figs. S4 and S5). These findings were further verified at the national level, with persistent gender differences (Tables S6, S7, and S8). The ASIR, ASMR, and ASDR among males were greater than those of females. However, these indicators for males are declining, whereas for females, they are rising rapidly, particularly in developing countries. In 2021, China ranked first globally in terms of the number of incident cases (172,394), deaths (428,031), and DALYs (6,752,287) (Tables S9, S10, and S11). Monaco presented the highest ASIR, ASMR, and ASDR. Globally, Monaco exhibited the most substantial increases in ASIR and ASMR, whereas Greenland experienced the most pronounced decrease (Figs. 5, S6 and S7).

### Attributable risk factors for DALY in older TBL cancer patients

In the GBD study, smoking, environmental particulate matter pollution, and occupational asbestos exposure were the major risk factors for DALYs due to TBL in both sexes, males, and females, in 2021 (Figs. S8, S9, and S10). Nevertheless, the proportion attributable to smoking has been declining, whereas that attributable to environmental particulate matter has been increasing (Fig. S11). Among all the SDI quintiles, household air pollution from solid fuels and a low-fruit diet contributed to the highest proportions of TBL cancer DALYs in low-SDI regions in 2021, accounting for 32.20% and 9.81%, respectively. In contrast, in high-SDI countries, these two factors contributed to the lowest proportions of TBL cancer DALYs, 0.03% and 2.67%, respectively. Supplementary Fig. 12 shows that in the 21 GBD regions, the proportions of DALYs caused by household air pollution from solid fuels and a low-fruit diet were negatively correlated with the SDI.

Compared with female patients, male patients with TBL-related cancer were more likely to be affected by most risk factors, particularly smoking. Notably, from 1990 to 2021, the proportions of DALYs attributable to smoking declined for males and females (Figs. S13 and S14), but with different magnitudes. The proportion of males decreased from 76.0% to 72.0% (a 4.0 percentage point decline), whereas the proportion of females decreased more significantly from 43.0% to 34.0% (a 9.0 percentage point decline), which was greater than that of males. Supplementary Figs. 15 and 16 further indicate that in the 21 regions covered by the GBD, the proportions of DALYs caused by smoking among male and female patients are positively correlated with the SDI. Notably, the proportion of DALYs caused by smoking among male patients started to decrease after reaching a peak at a relatively high SDI. Additionally, compared with male patients, female patients were more likely to be affected by secondhand smoke exposure and household air pollution, particularly in middle- and low-SDI regions (Figures S12 and S13). Supplementary Fig. 16 indicates that among the female patients across the 21 regions covered by the GBD study, the proportion of DALYs attributable to second-hand smoke exposure tended to increase with increasing SDI. It reached its peak at the medium–high SDI level and then started to decline. In contrast, the proportion of DALYs caused by household air pollution was significantly negatively correlated with the SDI.

### Forecast of the burden of older TBL cancer patients by 2035

**Fig. 6:**
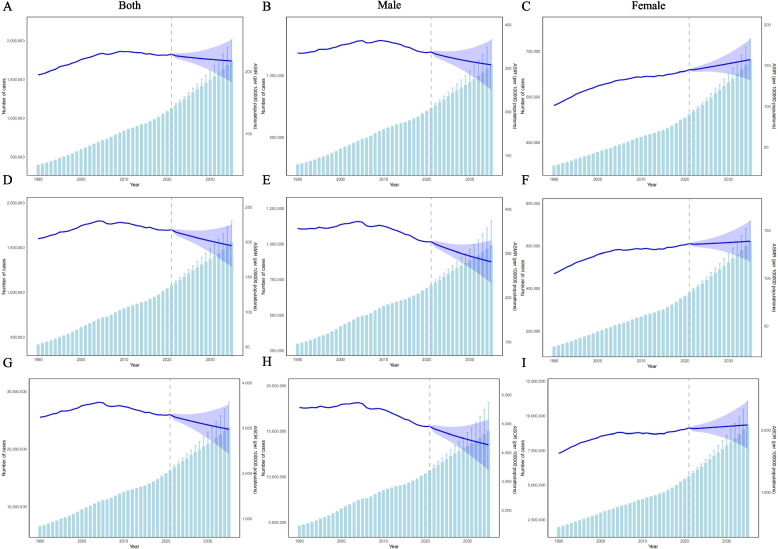
Projection of trends regarding TBL cancer conditions in older patients (aged 70 years and older) from 2021 to 2035, calculated by BAPC models. Trends in the incidence of both sexes (**A**), males (**B**), and females (**C**); deaths of both sexes (**D**), males (**E**), and females (**F**); and the DALYs of both sexes (**G**), males (**H**), and females (**I**). Abbreviations: ASIR = age-standardized incidence rate, ASMR = age-standardized mortality rate, ASDR = age-standardized rate of DALYs, DALYs = disability-adjusted life years, BAPC = Bayesian age‐period‐cohort, TBL = tracheal, bronchial, and lung

The prediction model revealed that between 2022 and 2035, the global incidence, mortality, and DALY rates are projected to rise. Specifically, the estimated numbers are expected to reach 1,737,986 cases (95% CI: 1,463,365–2,012,607), 1,559,570 cases (95% CI: 1,316,431–1,802,708), and 23,803,965 cases (95% CI: 19,453,610–28,154,320), respectively. Moreover, we projected trends in the ASIR, ASMR, and ASDR for older TBL cancer patients after 2021 stratified by sex. The ASIR, ASMR, and ASDR in both sexes and among male patients with TBL-related cancer are projected to decrease (Fig. 6). Conversely, for females, the ASIR, ASMR, and ASDR are expected to remain relatively stable or increase, increasing from 144.77 per 100,000, 136.25 per 100,000, and 2036.81 per 100,000 population in 2021 to 157.25 per 100,000, 138.77 per 100,000, and 2085.27 per 100,000 population by 2035, respectively (Figs. 6C, F, and I and Table S12).

## Discussion

This study is the first global systematic assessment of the burden of and risk factors for TBL cancer among patients aged 70 years and above from 1990 to 2021. The results revealed that from 1990 to 2021, the ASIR, ASMR, and ASDR of lung cancer globally demonstrated an overall increasing trend. Decomposition analyses indicated that this trend was consistent with the acceleration of global population aging and the well-established association between TBL-related cancer and age. However, further analysis across different lung cancer treatments revealed that the aforementioned indicators started to decline during the targeted therapy breakthrough era (2004–2014), and both the ASMR and ASDR accelerated the decline during the precision immunotherapy era (2015–2021). Predictive analysis indicated that the number of incident cases, deaths, and DALY cases among elderly lung cancer patients will continue to rise over the next 14 years. Despite significant improvements in patient prognosis due to treatment advancements, the disease burden of lung cancer has persisted. This phenomenon may be related to persistent risk factors in the TBL cancer population and the uneven distribution of medical resources.

In 2003, the FDA approved gefitinib as the first targeted drug for the treatment of NSCLC. Since 2004, this drug has been widely adopted in clinical practice. This event marked the beginning of the targeted therapy era with EGFR-TKIs, representing an important milestone in the field of lung cancer treatment [9]. Subsequently, the treatment of lung cancer has shifted from the traditional one-size-fits-all model of surgery and chemotherapy to an era of more precise, tailor-made targeted therapy. In 2011, the FDA approved crizotinib for the treatment of patients with anaplastic lymphoma kinase (ALK)-positive NSCLC. This was another major breakthrough in the field of targeted therapy for lung cancer, further expanding the scope of the application of targeted therapy [26]. Recent clinical studies have shown that first-line sequential treatment with crizotinib can prolong the median overall survival of patients to more than 7 years [27]. Despite the rapid development of targeted drugs, there have been few trials specifically focusing on their efficacy in elderly patients [4, 28]. Therefore, our study further focused on the temporal trends of the ASMR and ASDR among global lung cancer patients aged 70 and above before and after 2004. Joinpoint regression analysis showed that the above—mentioned indicators continued to rise before 2004 and continued to decline after 2004. This further indicates that the continuous development of targeted therapy and the widespread application of molecular detection methods have played a positive and significant role in improving the prognosis of elderly patients.

In 2015, the FDA approved the use of programmed PD-1 monoclonal antibodies for the treatment of lung cancer. This groundbreaking decision undoubtedly marked another crucial milestone in the revolutionary journey of lung cancer treatment, opening a new chapter in this field. In recent years, the multidimensional precision treatment model of immunotherapy combination has emerged, bringing new hope and a glimmer of dawn for the long-term survival of lung cancer patients [20]. The findings of a cohort study based on the large-scale Surveillance, Epidemiology, and End Results (SEER) database revealed that immunotherapy can significantly improve the prognosis of lung cancer patients [29]. However, this study was only conducted in an American population, and patients from other countries were not included. Consequently, our study further focused on the global elderly lung cancer patient population from 2015 to 2021. The results revealed that during this period, both the ASMR and ASDR of elderly lung cancer patients significantly declined, with AAPCs of −0.59 (95% CI: −0.92, −0.26, *p* < 0.05) and −0.29 (−0.50, −0.12, *p* < 0.05), respectively. These results confirm the favorable and notable impact of precision immunotherapy on the prognosis of elderly patients.

In the era of booming precision therapy, a worrisome phenomenon in TBL cancer incidence has emerged: the ASIR of lung cancer is decreasing in males but increasing in females. Since 2004, the growth of the ASIR among elderly male TBL cancer patients has slowed, peaking in 2011 and then declining. This trend is likely associated with public health policies. In 2003, 182 countries adopted the landmark Framework Convention on Tobacco Control, aiming to reduce tobacco-related diseases and mortality through measures such as advertising restrictions and tax hikes [30]. In contrast, the ASIR of lung cancer among elderly females showed a significant upward trend in all three eras of lung cancer treatment. Part of the reason for this phenomenon may be that elderly females are more likely to continue smoking than males [31]. It is also possible that because females make different judgments on smoking in terms of social connection and weight management, females generally do not respond as actively as males to tobacco control intervention measures [32]. Additionally, our study revealed that the proportion of DALYs caused by smoking among male and female patients tended to increase with increasing SDI. However, among male patients, this proportion began to decline after the SDI reached a high level. This phenomenon underscores the need for public health interventions tailored to different sexes, particularly measures for females that align with their needs and characteristics to increase tobacco control support.

Our study revealed that during the era of precision immunotherapy (2015–2021), the ASMRs and ASDRs among female patients tended to increase, whereas those among male patients tended to decrease, further widening the gap between the sexes. This phenomenon may be ascribed to multiple factors, including sociocultural, environmental, and biological factors.

Firstly, sociocultural factors have intensified health inequalities to a certain degree. Elderly female patients have a greater proportion of DALYs caused by exposure to household air pollution than male patients, especially in regions with low-SDI regions. Previous studies have indicated that elderly female patients encounter more obstacles in accessing medical resources, leading to increases in their physical disability and mortality rates [33]. This situation contributes to widening the health gap between genders.

Secondly, biological factors also play a crucial role. The risk factor analysis in our study revealed that the proportion of smokers among male patients was significantly greater than that among female patients. Previous studies have shown that smoking can increase the tumor mutation burden (TMB) and promote high expression of PD-L1 [34]. Both factors are more likely to activate the immune response. A prospective study by Wang et al. with 644 patients revealed that increased smoking duration was significantly associated with better clinical outcomes of immune checkpoint inhibitor monotherapy (OR, objective response rate [ORR] = 1.21, 95% CI: 1.09–1.36, *P* < 0.001; HR, overall survival [OS] = 0.94, 95% CI: 0.90–0.99, *P* = 0.01) [35]. Therefore, male patients may experience better efficacy in immunotherapy than female patients, which further widens the health gap between the sexes in the era of precision immunotherapy. A meta-analysis by Conforti and colleagues also revealed that the efficacy of immunotherapy in men is significantly better than that in women (*P* = 0.002) [36].

Extended BAPC modeling reveals divergent epidemiological trajectories: the incidence of TBL cancer among elderly males is projected to decrease by 2035, whereas elderly females may experience an 8.59% relative increase in disease burden over the same fourteen-year period. Future TBL cancer prevention and control strategies may need to go beyond the traditional biomedical model. A multidimensional intervention system encompassing social (e.g., better access to medical resources for women), behavioral (e.g., smoking cessation), environmental (e.g., air pollution reduction), and physiological aspects might be needed to address different epidemiological trends among elderly males and females.

Age group analysis revealed that the incidence of TBL cancer in patients aged ≥ 75 years, as well as the mortality rate and DALY among those aged ≥ 80 years, increased, indicating significant differences in disease incidence across age groups. As the worldwide aging phenomenon has accelerated, the increasing percentage of older adults has contributed significantly to the increase in TBL cancer incidence and mortality [37]. This finding is consistent with our results, highlighting the continually increasing incidence, mortality, and DALY burden of TBL cancer among older individuals. Therefore, when developing screening and treatment strategies, it is essential to fully consider patient life expectancy and health status to optimize resource allocation and improve patient prognosis.

Our research revealed that from 1990 to 2021, the ASMRs and ASDRs among those aged ≥ 70 years declined in high-SDI regions but increased in middle- and low-SDI regions. Interestingly, there was a negative correlation between the AAPC and SDI scores. This phenomenon may be due to weaker health care systems in low-SDI countries or regions, where a lack of essential medical resources makes it difficult for lung cancer patients to receive timely diagnosis and treatment [38]. Additionally, the economic conditions in these areas limit older individuals’ ability to afford medical expenses, thereby increasing the risk of disability and mortality from TBL cancer among older individuals [39]. Although economic development is rapid in countries with middle-SDI, the population’s health awareness remains relatively low. Unhealthy living habits and extensive environmental pollution have jointly contributed to a greater cancer burden in older TBL patients [40–42]. Our research results also indicated that household air pollution from solid fuels and low fruit intake were major risk factors for the DALYs of TBL cancer in low- and low-middle-SDI countries in 2021, whereas these factors were not significant in middle-, middle-high-, and high-SDI countries. However, areas with high SDI levels have significantly reduced the burden of lung cancer among older individuals through well-established health care systems, heightened health awareness, and strict public health policies such as smoking cessation programs and early diagnostic screenings [43, 44]. This phenomenon provides actionable insights for public health officials in middle-SDI regions to combat rising TBL cancer rates.

Our research suggested that regional-level interventions are crucial in regions such as East Asia and western sub-Saharan Africa, where TBL cancer incidence, mortality, and DALYs among older people have increased sharply. In East Asia, population aging plays a significant role in the increasing mortality rates associated with age-related cancers [45]. In sub-Saharan West Africa, the substantial burden of TBL cancer is attributed primarily to limited access to health care services and the absence of effective early detection and treatment programs [46]. Furthermore, our research findings indicate that although environmental particulate pollution is a global issue, its impact is disproportionately concentrated in regions with middle, low-middle, and low SDI levels, in contrast to high-SDI countries. This finding is consistent with a recent study showing that approximately 7.3 billion people worldwide are exposed to unsafe levels of PM2.5, with 80% residing in less developed or middle-income countries, such as East Asia and sub-Saharan Africa [47]. Thus, public health efforts in these areas should focus on enhancing early screening program coverage, bolstering health care infrastructure, and taking action against regional risks such as air pollution and unhealthy habits. In high-income areas with decreasing incidence rates and DALYs, such as high-income North America and Central Latin America, sustaining investments and optimizing existing health care programs are vital to address population aging and evolving risks.

At the national level, in 2021, China had the highest TBL cancer ASIR, ASMR, and ASDR among older people globally, which aligns with findings from other regions. Moreover, Monaco exhibited the fastest increase in TBL cancer incidence among senior patients. These results highlight the urgent need to formulate national policies to increase health awareness among older people and to strengthen screening efforts [48]. Overall, the findings from these regions and countries align with the global strategic goal of reducing lung cancer mortality while also underscoring the importance of continuously evaluating and adjusting policies to address evolving trends in TBL cancer incidence and mortality.

This study has several limitations. First, the quality of the data may be affected by incomplete disease management systems in different countries and regions, particularly due to the lack of reliable data from low-income countries. Second, the GBD database does not distinguish lung cancer pathological subtypes and lacks molecular testing data, as lung cancer subtypes and molecular mutation characteristics vary across regions [49]. Moreover, there is an absence of clinical data regarding patient treatment, which makes it difficult to conduct more in-depth analysis. Therefore, future studies with larger sample sizes and more extensive data are needed for further verification.

## Conclusions

In conclusion, this study comprehensively examined the global TBL cancer burden in elderly individuals from 1990–2021, revealing increases in the ASIRs, ASMRs, and ASDRs. During the precision immunotherapy era (2015–2021), health disparities widened among both males and females. High-SDI countries have experienced a reduced burden, whereas it has increased in middle-SDI countries, highlighting the impact of socioeconomic factors. By 2035, the number of cases of TBL cancer among the elderly will increase further. Therefore, in the era of precision medicine, it could be advisable to incorporate more elderly individuals into clinical trials and to formulate management strategies for elderly TBL cancer patients. Potential approaches to alleviate the TBL cancer burden among elderly individuals could include strengthening smoking cessation programs for elderly women, enhancing air pollution control, expanding the coverage of early screening programs in low-SDI regions, and improving the accessibility of precision treatments.

## Supplementary Information

Supplementary Material 1

Supplementary Material 2: Figure S1. Association between older TBL cancer patients (aged 70 years and older) with ASIRs, ASDRs, and ASMRs and SDIs in 204 countries and territories from 1990 to 2021. SDI vs ASIRs: (A) both sexes, (B) males, (C) females; SDI vs ASMRs: (D) both sexes, (E) males, (F) females; SDI vs ASDRs: (G) both sexes, (H) males, (I) females. Abbreviations: ASIR = age-standardized incidence rate, ASMR = age-standardized mortality rate, ASDR = age-standardized rate of DALYs, DALYs = disability-adjusted life years, SDI = sociodemographic index, TBL = tracheal, bronchial, and lung. Figure S2. AAPCs of the ASIR (A), ASMR (B), and ASDR (C) from 1990 to 2021 in 204 countries and territories according to the SDI in 2021. Abbreviations: ASIR = age-standardized incidence rate, ASMR = age-standardized mortality rate, ASDR = age-standardized rate of DALYs, AAPCs = average annual percent changes, DALYs = disability-adjusted life years, SDI = sociodemographic index, TBL = tracheal, bronchial, and lung. Figure S3. Comparison of the ASIR, ASMR, and ASDR for older TBL cancer patients (aged 70 years and older) in both sexes across 21 geographical GBD regions by the SDI for 1990, 2004, 2015 and 2021. (A) ASIR, (B) ASMR, (C) ASDR. Abbreviations: ASIRs = age-standardized incidence rate, ASMRs = age-standardized mortality rate, ASDRs = age-standardized rate of DALYs, DALYs = disability-adjusted life years, GBD = global burden of disease, SDI = socialdemographic index, TBL = tracheal, bronchial, and lung. Figure S4. Comparison of the ASIR, ASMR, and ASDR for older TBL cancer patients (aged 70 years and older) in male across 21 geographical GBD regions by the SDI for 1990, 2004, 2015 and 2021. (A) ASIR, (B) ASMR, (C) ASDR. Abbreviations: ASIRs = age-standardized incidence rate, ASMRs = age-standardized mortality rate, ASDRs = age-standardized rate of DALYs, DALYs = disability-adjusted life years, GBD = global burden of disease, SDI = socialdemographic index, TBL = tracheal, bronchial, and lung. Figure S5. Comparison of the ASIR, ASMR, and ASDR for older TBL cancer patients (aged 70 years and older) in female across 21 geographical GBD regions by the SDI for 1990, 2004, 2015 and 2021. (A) ASIR, (B) ASMR, (C) ASDR. Abbreviations: ASIRs = age-standardized incidence rate, ASMRs = age-standardized mortality rate, ASDRs = age-standardized rate of DALYs, DALYs = disability-adjusted life years, GBD = global burden of disease, SDI = socialdemographic index, TBL = tracheal, bronchial, and lung. Figure S6. The global distribution of the ASIR, ASMR, and ASDR of TBL cancer in older male patients (aged 70 years and older) in 1990 and 2021. Global maps of ASIR (A), ASMR (B), and ASDR (C) in 1990; and global maps of ASIR (D), ASMR (E), and ASDR (F) in 2021. Abbreviations: ASIR = age-standardized incidence rate, ASMR = age-standardized mortality rate, ASDR = age-standardized rate of DALYs, DALYs = disability-adjusted life years, TBL = tracheal, bronchial, and lung. Figure S7. The global distribution of the ASIR, ASMR, and ASDR of TBL cancer in older female patients (aged 70 years and older) in 1990 and 2021. Global maps of ASIR (A), ASMR (B), and ASDR (C) in 1990; and global maps of ASIR (D), ASMR (E), and ASDR (F) in 2021. Abbreviations: ASIR = age-standardized incidence rate, ASMR = age-standardized mortality rate, ASDR = age-standardized rate of DALYs, DALYs = disability-adjusted life years, TBL = tracheal, bronchial, and lung. Figure S8. Proportion of TBL cancer in both sexes older patients (aged 70 years and older) DALYs attributable to 16 risk factors globally and classified by SDI levels in 2021. Abbreviations: DALYs = disability-adjusted life years, TBL = tracheal, bronchial, and lung, SDI = sociodemographic index. Figure S9. Proportion of TBL cancer in older male patients (aged 70 years and older) DALYs attributable to 16 risk factors globally and classified by SDI levels in 2021. Abbreviations: DALYs = disability-adjusted life years, TBL = tracheal, bronchial, and lung, SDI = sociodemographic index. Figure S10. Proportion of TBL cancer in older female patients (aged 70 years and older) DALYs attributable to 16 risk factors globally and classified by SDI levels in 2021. Abbreviations: DALYs = disability-adjusted life years, TBL = tracheal, bronchial, and lung, SDI = sociodemographic index. Figure S11. Global ASIR, ASMR and ASDR for TBL cancer among older patients for both sexes by SDI, 1990-2021. Abbreviations: ASIR = age-standardized incidence rate, ASMR = age-standardized mortality rate, ASDR = age-standardized rate of DALYs, DALYs = disability-adjusted life years, TBL = tracheal, bronchial, and lung, SDI = sociodemographic index. Figure S12. Association between SDI and the proportions of DALYs attributable to 16 risk factors for TBL cancer among older patients for both sexes in 21 GBD regions, 2021. Abbreviations: DALYs = disability-adjusted life years, TBL = tracheal, bronchial, and lung, SDI = sociodemographic index, GBD = global burden of disease. Figure S13. Global ASIR, ASMR and ASDR for TBL cancer among older patients for female by SDI, 1990-2021. Abbreviations: ASIR = age-standardized incidence rate, ASMR = age-standardized mortality rate, ASDR = age-standardized rate of DALYs, DALYs = disability-adjusted life years, TBL = tracheal, bronchial, and lung, SDI = sociodemographic index. Figure S14. Global ASIR, ASMR and ASDR for TBL cancer among older patients for male by SDI, 1990-2021. Abbreviations: ASIR = age-standardized incidence rate, ASMR = age-standardized mortality rate, ASDR = age-standardized rate of DALYs, DALYs = disability-adjusted life years, TBL = tracheal, bronchial, and lung, SDI = sociodemographic index. Figure S15. Association between SDI and the proportions of DALYs attributable to 16 risk factors for TBL cancer among older patients for male in 21 GBD regions, 2021. Abbreviations: DALYs = disability-adjusted life years, TBL = tracheal, bronchial, and lung, SDI = sociodemographic index, GBD = global burden of disease. Figure S16. Association between SDI and the proportions of DALYs attributable to 16 risk factors for TBL cancer among older patients for female in 21 GBD regions, 2021. Abbreviations: DALYs = disability-adjusted life years, TBL = tracheal, bronchial, and lung, SDI = sociodemographic index, GBD = global burden of disease.

## Data Availability

The data used in this study came from a public database that everyone can access through the link provided in this article (https://vizhub.healthdata.org/gbd-results/). The source code for this project is available at https://ghdx.healthdata.org/gbd-2021/code.

## Abbreviations

AAPCs: Average annual percent changes
APC: Annual percentage changes
ASDRs: Age-standardized rate of DALYs
ASIRs: Age-standardized incidence rates
ASMRs: Age-standardized mortality rates
BAPC: Bayesian age‒period‒cohort
DALYs: Disability-adjusted life years
EORTC: European Organization for Research and Treatment of Cancer
FDA: Food and Drug Administration
GBD: Global Burden of Disease
SIOG: International Society of Geriatric Oncology
NSCLC: Non-small cell lung cancer
SDI: Sociodemographic index
TBL: Tracheal, bronchial, and lung
UIs: Uncertainty intervals
