# Supplementary material for: Tracheal, bronchus, and lung cancer among older adults: thirty-year global burden trends, precision medicine breakthroughs, and lingering barriers
Source: BMC Cancer. 2025 May 28;25:954. doi: 10.1186/s12885-025-14363-x (PMC12117747; doi:10.1186/s12885-025-14363-x)
Supplement: Supplementary file 2 — Supplementary Material 2: Figure S1. Association between older TBL cancer patients (aged 70 years and older) with ASIRs, ASDRs, and ASMRs and SDIs in 204 countries and territories from 1990 to 2021. SDI vs ASIRs: (A) both sexes, (B) males, (C) females; SDI vs ASMRs: (D) both sexes, (E) males, (F) females; SDI vs ASDRs: (G) both sexes, (H) males, (I) females. Abbreviations: ASIR = age-standardized incidence rate, ASMR = age-standardized mortality rate, ASDR = age-standardized rate of DALYs, DALYs = disability-adjusted life years, SDI = sociodemographic index, TBL = tracheal, bronchial, and lung. Figure S2. AAPCs of the ASIR (A), ASMR (B), and ASDR (C) from 1990 to 2021 in 204 countries and territories according to the SDI in 2021. Abbreviations: ASIR = age-standardized incidence rate, ASMR = age-standardized mortality rate, ASDR = age-standardized rate of DALYs, AAPCs = average annual percent changes, DALYs = disability-adjusted life years, SDI = sociodemographic index, TBL = tracheal, bronchial, and lung. Figure S3. Comparison of the ASIR, ASMR, and ASDR for older TBL cancer patients (aged 70 years and older) in both sexes across 21 geographical GBD regions by the SDI for 1990, 2004, 2015 and 2021. (A) ASIR, (B) ASMR, (C) ASDR. Abbreviations: ASIRs = age-standardized incidence rate, ASMRs = age-standardized mortality rate, ASDRs = age-standardized rate of DALYs, DALYs = disability-adjusted life years, GBD = global burden of disease, SDI = socialdemographic index, TBL = tracheal, bronchial, and lung. Figure S4. Comparison of the ASIR, ASMR, and ASDR for older TBL cancer patients (aged 70 years and older) in male across 21 geographical GBD regions by the SDI for 1990, 2004, 2015 and 2021. (A) ASIR, (B) ASMR, (C) ASDR. Abbreviations: ASIRs = age-standardized incidence rate, ASMRs = age-standardized mortality rate, ASDRs = age-standardized rate of DALYs, DALYs = disability-adjusted life years, GBD = global burden of disease, SDI = socialdemographic index, TBL = tracheal [file 12885_2025_14363_MOESM2_ESM.zip › Figure S9.pdf]

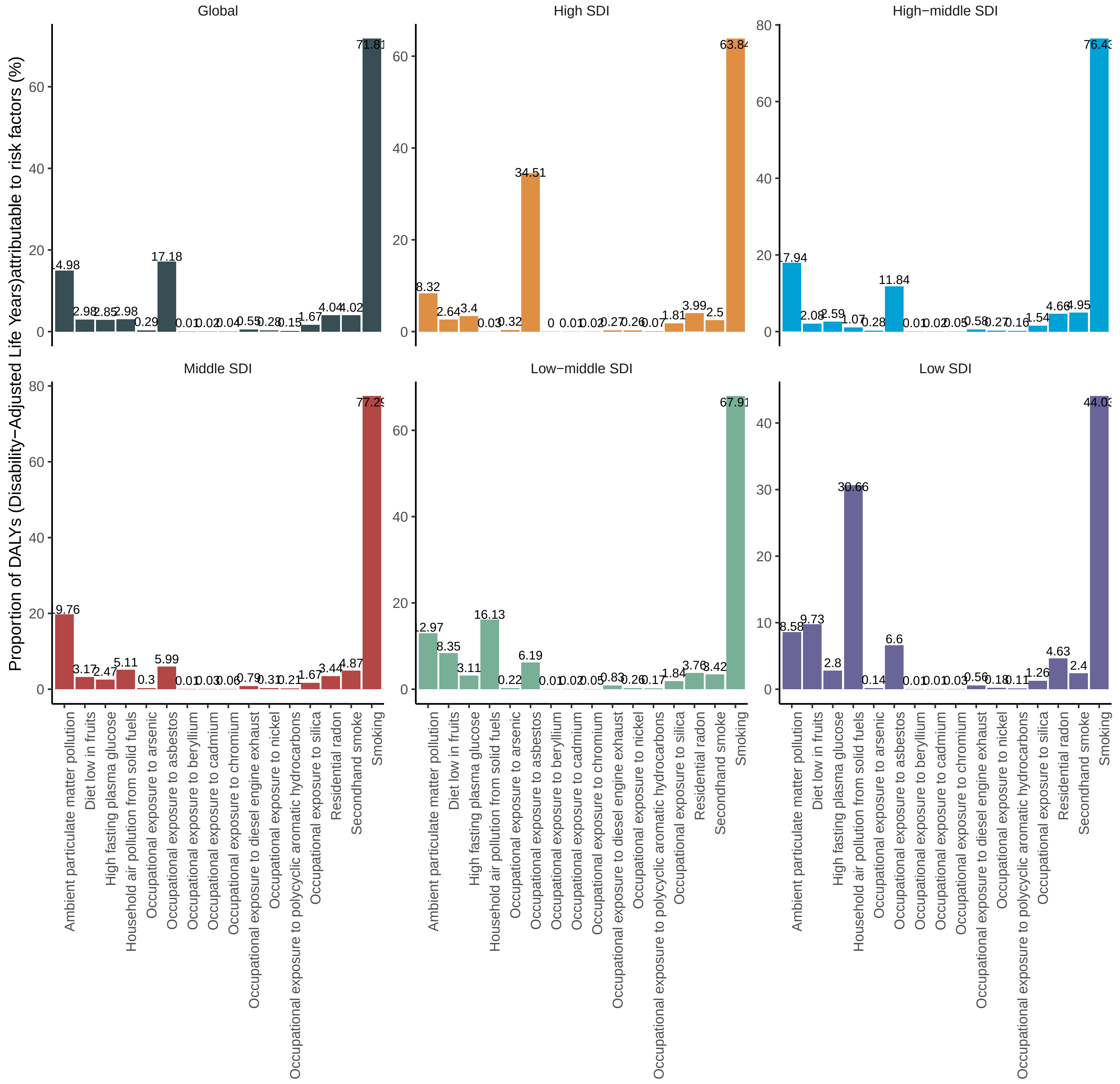

Global
High SDI
High−middle SDI
Middle SDI
Low−middle SDI
Low SDI
Proportion of DALYs (Disability−Adjusted Life Years)attributable to risk factors (%)
Ambient particulate matter pollution
Diet low in fruits
High fasting plasma glucose
Household air pollution from solid fuels
Occupational exposure to arsenic
Occupational exposure to asbestos
Occupational exposure to beryllium
Occupational exposure to cadmium
Occupational exposure to chromium
Occupational exposure to diesel engine exhaust
Occupational exposure to nickel
Occupational exposure to polycyclic aromatic hydrocarbons
Occupational exposure to silica
Residential radon
Secondhand smoke
Smoking
